# Hypoglycemic Effect of Edible Fungi Polysaccharides Depends on Their Metabolites from the Fermentation of Human Fecal Microbiota

**DOI:** 10.3390/foods13010097

**Published:** 2023-12-27

**Authors:** Rongxuan Yu, Jianming Luo, Liu Liu, Xichun Peng

**Affiliations:** Department of Food Science and Engineering, Jinan University, Guangzhou 510632, China

**Keywords:** edible fungi polysaccharides, in vitro fermentation, hypoglycemic, metabolic profiles, key metabolites, IR-HepG2 cells

## Abstract

Edible fungi polysaccharides are widely sourced and have various physiological activities, including hypoglycemic. Current studies mainly focus on the hypoglycemic activity of polysaccharides themselves, while the strength of the hypoglycemic activity of edible fungi polysaccharides from different sources remained elusive. This study compared the hypoglycemic activity of different edible fungi polysaccharides after in vitro fermentation by fecal bacteria, combined with non-targeted metabolomics and 16S rDNA analysis, to screen out potential key metabolites related to the hypoglycemic activity. The results show that the fermentation supernatants of all four edible fungi polysaccharides significantly increased the glucose consumption and glycogen synthesis of IR-HepG2, also up-regulated the level of hexokinase and down-regulated the level of phosphoenolpyruvate carboxylase. All fermentation supernatants could alleviate the insulin resistance of IR-HepG2 cells by regulating the expression levels of genes related to the IRS-1/PI3K/Akt signaling pathway. Gingerglycolipid A, sphinganine 1-phosphate, matricin, tricarballylic acid, N-carbamoylputrescine, nomega-acetylhistamine, tyramine, and benzamide could be considered as potential key metabolites to evaluate the hypoglycemic effects. Their levels were strongly positively correlated with the abundance of *Candidatus_Stoquefichu*, *Faecalibacterium*, *Coprococcus*, *Bacteroides*, *Eubacterium_ventriosum_group*, *Anaerostipes*, *Parabacteroides*, and *Agathobacter*. These metabolites and microorganisms are closely related to the hypoglycemic activity of edible fungi polysaccharides.

## 1. Introduction

Diabetes is a metabolic disease due to the body’s inability to produce insulin or its inability to fully utilize insulin. Data from the International Diabetes Federation showed that there will be 537 million diabetics between the ages of 20 and 79 by 2021, accounting for 10.5% of the global population in this age group, and this number is projected to rise to 12.2% in 2045 [1]. Diabetes has become one of the most serious global public health threats of our time. However, most of the hypoglycemic drugs currently used to treat diabetes, such as metformin, have limitations and some side effects [2]. It is worth noting that most diabetes may be caused by unhealthy dietary habits [3], indicating that daily prevention and therapy are equally important. Therefore, natural active ingredients of plants have become good candidates for the prevention and treatment of diabetes due to their diverse bioactivities, lesser side effects, and low cost.

Edible fungi contain a variety of bioactive ingredients with nutritional and pharmaceutical value. Recent studies have shown that they exhibit significant hypoglycemic activity and can be used as effective dietary supplements to prevent and improve diabetes [4,5]. As the main active ingredient in edible fungi, polysaccharides have been proven to have hypoglycemic effects [6,7]. However, most edible fungi polysaccharides usually exert probiotic effects not by being directly digested and absorbed by the host but by being utilized as a carbon source of colonic microbiota [8]. During the colonic microbiota fermenting polysaccharides, the composition of colonic microbiota will change, such as the increasing abundance of probiotics [9]. Meanwhile, a large number of metabolites, such as short-chain fatty acids (SCFAs), are produced [10]. These fermentation metabolites of polysaccharides exhibit a variety of biological activities and play a role in alleviating and improving diabetes. An example is *Atractylodes chinensis* (DC). Koidz polysaccharide may alleviate type 2 diabetic symptoms by regulating intestinal microbiota and metabolites [11]. Inulin-type fructans showed the potential to improve gut microbiota composition in patients with type 2 diabetes and induced increased concentrations of fecal SCFAs [12]. Therefore, we speculate that polysaccharides will produce a large number of specific metabolites with hypoglycemic activity after being utilized by fecal microbiota. The levels of those specific metabolites will be responsible for the hypoglycemic effect of different polysaccharides.

Therefore, this research aimed to correlate the hypoglycemic effects of edible fungi polysaccharides with the levels of their intestinal metabolites or the abundance of related bacteria in order to find some potential key metabolites and bacteria that can be applied to evaluate the hypoglycemic activity of different polysaccharides.

## 2. Materials and Methods

### 2.1. Reagents and Materials 

*Ganoderma lucidum*, *Cordyceps militaris*, *Lentinula edodes* and *Poria cocos*, were purchased from Beijing Tongrentang Co., Ltd. (Beijing, China). HepG2 cells, fetal bovine serum (FBS), metformin (MET) and insulin were purchased from Procell Biotechnology Co., Ltd. (Wuhan, China). Dulbecco’s Modified Eagle’s Medium (DMEM) was purchased from Gibco BRL (Grand Island, NY, USA). The glucose assay kit, cell glycogen assay kit, hexokinase assay kit (HK), phosphoenolpyruvate carboxylase kinase kit (PEPCK), and protein concentration detection assay kit (BCA methods) were bought from Nanjing Jiancheng Co., Ltd. (Nanjing, China). TRIzol reagent was purchased from Thermo Fisher Scientific (Waltham, MA, USA). ChamQ Universal SYBR qPCR Master Mix was purchased from Nanjing Vazyme Biotech Co., Ltd. (Nanjing, China). All other chemicals and reagents used in this study were of the analytical reagent quality.

### 2.2. Preparation of Crude Polysaccharide

*Ganoderma lucidum*, *Cordyceps militaris* and *Lentinula edodes* were powdered and soaked in ethanol overnight for decolorization and degreasing. The decolorized and degreasing powder was extracted twice with distilled water at a ratio of 1:20, at 100 °C for 120 min each time. The supernatants of the two extractions were combined, then 0.5% neutral protease (*W*/*V*) was added to the supernatant and treated at 50 °C for 4 h. Residual proteins were removed using the Sevag method [13], followed by organic reagents using a vacuum evaporator. The supernatant was concentrated at four times the volume of absolute ethanol and was added for 12 h to precipitate the crude polysaccharide. The collected precipitates were redissolved in water and dialyzed for 48 h using a dialysis membrane with a molecular cutoff of 10,000 KDA. Finally, the crude *Ganoderma lucidum*, *Cordyceps militaris*, and *Lentinula edodes* polysaccharides (GLP, CMP, LEP) were obtained by vacuum freeze-drying.

The alkaline-soluble polysaccharide of *Poria cocos* was extracted according to Zhao’s method with slight modifications [14]. *Poria cocos* was powdered and soaked in ethanol overnight for decolorization and degreasing. The decolorized and degreasing powder was extracted twice with distilled water at a ratio of 1:20, at 100 °C for 120 min each time. Then, the precipitates were collected and extracted with 1 mol/L sodium hydroxide solution at a ratio of 1:60 for 60 min. The supernatant was collected and adjusted to pH 7 using 1 mol/L hydrochloric acid solution, then dialyzed for 48 h using a dialysis membrane with a molecular cutoff of 10,000 KDA. Finally, the crude *Poria cocos* alkaline-soluble polysaccharides (PCP) were obtained by vacuum freeze-drying.

### 2.3. In Vitro Fermentation of Fecal Inocula

In vitro fermentation of human feces was performed according to an established method with minor modifications [15]. The composition of the basic nutrient growth medium is: yeast extract (2.0 g/L), peptone (2.0 g/L), bile salts (0.5 g/L), hemin (0.02 g/L), L-cysteine (0.5 g/L), NaHCO_3_ (2.0 g/L), NaCl (0.1 g/L), CaCl_2_·6H_2_O (0.01 g/L), MgSO_4_·7H_2_O (0.01 g/L), K_2_HPO_4_ (0.04 g/L), KH_2_PO_4_ (0.04 g/L), vitamin K1 (0.01 g/L), resazurin (0.01 g/L), and Tween-80 (2.0 mL/L).

Fresh fecal samples were collected from healthy adult male volunteers who did not have gastrointestinal disease or had taken antibiotics and probiotic products within three months before the experiment. Fecal samples and basal fermentation medium were mixed for further use (fecal samples: basal fermentation medium = 1:9, *W*/*V*). Then, the mixture was filtered through a 100-mesh sieve to obtain feces homogenate. The fecal homogenate was added to the basic fermentation medium until the content reached 10%, then GLP, LEP, CMP and PCP were respectively added to the fermentation medium until the concentration of the polysaccharides to 10.0 g/L (*W*/*V*). All the above operations were performed in an anaerobic operating platform. Meanwhile, the negative control group used a basic fermentation medium without an added carbon source. The fermentation process lasted 8 h. During the fermentation process, filtered sterilized N_2_ was continuously supplied, and an anaerobic fermentation device was used to maintain the fermentation conditions at 37 °C and pH 7.0 ± 0.3.

The fermentation broth of each group was collected and named according to the fermentation time as 0 h negative control (CON-0), 0 h *Ganoderma lucidum* polysaccharide (GLP-0), 0 h *Poria cocos* alkaline-soluble polysaccharide (PCP-0), 0 h *Cordyceps militaris* polysaccharide (CMP-0), 0 h *Lentinula edodes* polysaccharide (LEP-0), 8 h negative control (CON-8), 8 h *Ganoderma lucidum* polysaccharide (GLP-8), 8 h *Poria cocos* alkaline-soluble polysaccharide (PCP-8), 8 h *Cordyceps militaris* polysaccharide (CMP-8) and 8 h *Lentinula edodes* polysaccharide (LEP-8). The fermentation broth was centrifuged (12,000× *g*, 4 °C, 10 min). The precipitates and supernatants were collected and frozen at −80 °C, respectively. A portion of the supernatants were filtered with 0.22 μm MCE filter and frozen at −80 °C for subsequent experiments. Three separate samples were evaluated independently for each treatment.

The informed consent was obtained from all human subjects. The samples used were a mixture of fecal samples from three volunteers. Sample collection process involved in this study would not cause any physical, psychological, legal or informational risks to volunteers. Colonic flora is only a medium for fermenting polysaccharides in our research. The research content does not involve biomedical research directly related to human health. This research was started in December 2022, and the reference standard is the “Measures for Ethical Review of Biomedical Research Involving Humans” implemented by the Central People’s Government of the People’s Republic of China on 1 December 2016. This standard does not clearly state that the intestinal flora excreted by the human body are human biological samples.

### 2.4. Hypoglycemic Effect of Fermentation Supernatant on IR-HepG2 Cells

#### 2.4.1. Cell Culture and Viability

HepG2 cells were cultured in DMEM medium containing 10% fetal bovine serum, 1% penicillin and streptomycin, with culture conditions of 37 °C and 5% CO_2_. The passage was performed when the cell confluence was 80~90%. In order to evaluate the cell viability, HepG2 cells in the logarithmic growth phase were plated in 96-well plates at 20,000 cells per well. After 12 h of culture, the medium was replaced with DMEM medium containing fermentation supernatants dissolved in different concentrations (0, 12.5, 25, 50, 100, and 200 μg/L). After incubation for 24 h, the medium was replaced with 100 μL of 10% CCK-8 solution each well. The absorbance of each well was measured at a wavelength of 450 nm with a microplate reader after 2 h to calculate the cell viability of each group. The non-cytotoxic concentration was selected for subsequent experiments.

#### 2.4.2. Establishment of Insulin Resistance Model

The IR-HepG2 cell model was established as previously described with some modifications [16]. Briefly, HepG2 cells in the logarithmic growth phase were plated in 96-well plates at 20,000 cells per well for 12 h. Next, the medium was replaced with DMEM medium containing insulin dissolved in different concentrations (0, 0.2, 1, 5, 25, and 125 μg/L). After treating the cells for 24 h, 36 h, and 48 h, respectively, the glucose concentration of the culture supernatant was detected by glucose assay kit. The glucose consumption (mg) was calculated by the glucose concentration before and after treatment. The optimal conditions for establishing the IR-HepG2 model were determined based on glucose consumption.

#### 2.4.3. Glucose Consumption Measurement

After establishing IR-HepG2 according to the determined conditions, the cells were washed with FBS-free DMEM medium. An amount of 100 μL DMEM medium containing the optimal concentration of fermentation supernatants was added to treat the cells. The normal HepG2 cells served as the blank called the normal group. The negative controls were IR-HepG2 cells cultured in a medium renewed alone and called the model group. The positive controls were IR-HepG2 cells treated with a medium containing metformin (100 μg/mL) and called the Met group. The groups treated with fermentation supernatants were named after fermentation broths (GLP-0, LEP-0, CMP-0, PCP-0, GLP-8, LEP-8, CMP-8, and CON-8). After 24 h of incubation, the supernatants of the medium were collected to detect the glucose concentration using the glucose assay kit. The glucose consumption (mg) was calculated by the glucose concentration before and after treatment.

#### 2.4.4. Determination of Glycogen Content, Activity of Hexokinase (HK), Phosphoenolpyruvate Carboxylase Kinase (PEPCK)

IR-HepG2 cells were established and treated according to the method in Section 2.4.3. The treated IR-HepG2 cells were dispersed using trypsin and disrupted by sonication in a low-temperature environment. The HepG2 intracellular glycogen content and activities of HK and PEPCK in the normal, model, Met, and fermentation supernatant groups were determined according to the instructions of cell glycogen, HK, and PEPCK assay kits. The protein concentration of the disrupted cell homogenate of all groups was determined using the protein concentration detection assay kit (BCA methods).

#### 2.4.5. Quantitative Reverse Transcription Polymerase Chain Reaction

Total RNAs from IR-HepG2 cells were extracted using the TRIzol method. The extracted RNA samples were diluted and checked using NanoDrop2000. Subsequent experiments can only be carried out when the values of A260/A280 are within the range of 1.9 to 2.1. RNA was reverse transcribed into cDNA using HiScript^®^ II SuperMix for qPCR. The resulting cDNA was quantified by RT-qPCR using ChamQ Universal SYBR qPCR Master Mix according to the manufacturer’s instructions. The relative gene expression was normalized using GAPDH as an internal control and represented using the 2^−ΔΔCT^ method. Primers in this study are listed in Table 1. The CDS sequences of the primers used in quantitative real-time PCR were searched through the NCBI website, and the primers were designed by Sangon Bioengineering (Shanghai, China) Co., Ltd. All designed primers were verified through the Basic Local Alignment Search Tool on the NCBI website.

### 2.5. 16S rDNA Sequencing and Bioinformatics Analysis

Genomic DNA of all 8 h fermentation groups (CON-8, GLP-8, LEP-8, CMP-8, and PCP-8) and feces homogenate (OR) was determined by 16S rDNA sequencing. It should be noted that the microbial composition of the OR group was used to represent the microbial composition of the 0 h fermentation groups (GLP-0, LEP-0, CMP-0, PCP-0, and CON-0). Genomic DNA was extracted from each bacterial precipitation sample, and the V_3_-V_4_ region of the 16S rRNA was used as a bacterial-specific fragment with the primers 338F (5′-ACTCCTACGGGAGGCAGCAG-3′) and 806R (5′-GGACTACHVGGGTWTCTAAT-3′) [17]. Sequencing was performed by Shanghai Majorbio Bio-pharm Technology Co., Ltd. (Shanghai, China) [18]. USEARCH was used to perform cluster analysis on sequences with a similarity of 97% and define the sequences as operational taxonomic units (OTUs). Microbiota profiles of individual samples were analyzed following taxonomic analysis of representative OTU sequences.

### 2.6. Non-Targeted Metabolomics Analysis of Metabolite

Metabolite of 0 h fermentation groups (CON-0, GLP-0, LEP-0, CMP-0, and PCP-0) and 8 h fermentation groups (CON-8, GLP-8, LEP-8, CMP-8, and PCP-8) were analyzed by non-targeted metabolomic analysis. The LC-MS/MS analysis of the sample was conducted on a Thermo UHPLC-Q Exactive HF-X system equipped with an ACQUITYHSS T3 column (100 mm × 2.1 mm i.d., 1.8 μm; Waters, Milford, MA, USA) at Majorbio Bio-Pharm Technology Co., Ltd. (Shanghai, China). The mass spectrometric data were collected using a Thermo UHPLC-Q Exactive HF-X Mass Spectrometer equipped with an electrospray ionization (ESI) source operating in positive mode and negative mode. Data acquisition was performed using the Data Dependent Acquisition (DDA) mode. The detection was carried out over a mass range of 70–1050 *m*/*z*. Multivariate statistical analysis was performed using the ropls (Version 1.6.2) R package of Bioconductor on the Majorbio cloud platform (https://cloud.majorbio.com, accessed on 1 March 2023).

### 2.7. Statistical Analysis

Data are expressed as the means ± standard deviation calculated from triplicate experiments. SPSS 23.0 was used for statistical data analysis, and one-way analysis of variance and Duncan’s test were used for comparison between groups. Graphs were generated with GraphPad Prism 9. A correlation heatmap was generated using the Origin 2021 software.

## 3. Results

### 3.1. Effects of Fermentation Supernatants on Glucose Uptake in Insulin Resistance (IR) Cell Model

#### 3.1.1. Effect of Fermentation Supernatants on Cell Viability

**Figure 1:**
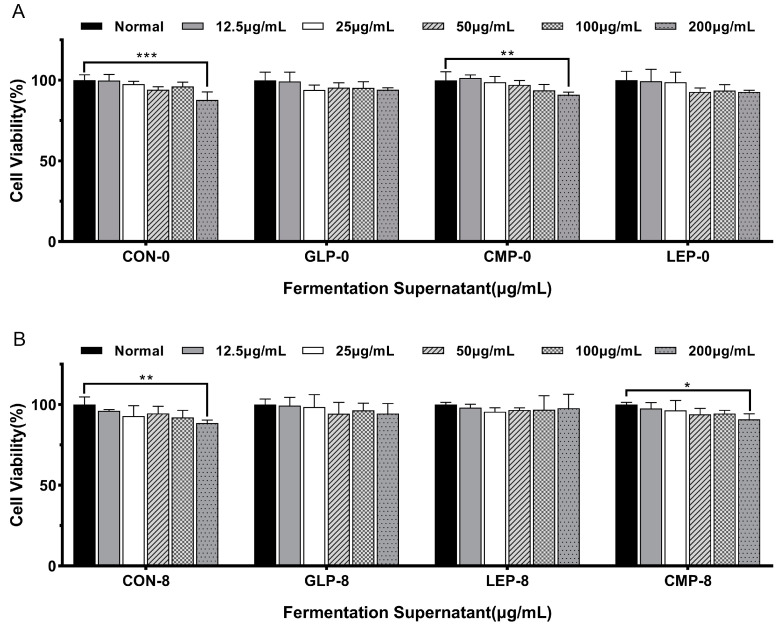
Effects of fermentation supernatants before and after fermentation of different polysaccharides on IR-HepG2 cell viability. (**A**) The cell viability in GLP-0, LEP-0, CMP-0, and CON-0 groups. (**B**) The cell viability in GLP-8, LEP-8, CMP-8, and CON-8 groups. Values are expressed as mean ± SD (n = 3). ‘*’ indicates *p* < 0.05, ‘**’ indicates *p* < 0.01, and ‘***’ indicates *p* < 0.001.

CCK-8 was used to evaluate the effect of the fermentation supernatants with different carbon sources and different fermentation times on HepG2 cell viability. As shown in Figure 1, the cell viability treated with fermentation supernatants from all treatment groups (GLP-0, LEP-0, CMP-0, CON-0, GLP-8, LEP-8, CMP-8, and CON-8) was greater than 90% when the fermentation supernatant concentration was less than 100 µg/mL, showing fermentation supernatants were not cytotoxic to HepG2 cells within this concentration range. Therefore, 100 µg/mL fermentation supernatant was selected for further investigation.

#### 3.1.2. Glucose Consumption in IR-HepG2

**Figure 2:**
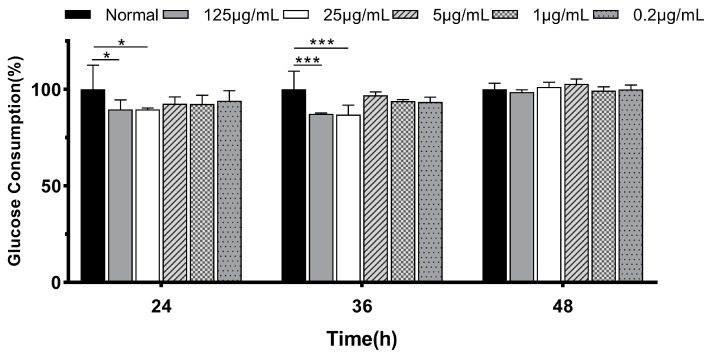
The glucose consumption in HepG2 cells is treated by insulin with different concentrations and at different times. Values are expressed as mean ± SD (n = 3). ‘*’ indicates *p* < 0.05 and ‘***’ indicates *p* < 0.001.

To understand the hypoglycemic effects of fermentation supernatants, we evaluated the glucose consumption in HepG2 cells. As shown in Figure 2, when cells were treated with 25 μg/mL insulin for 36 h, the glucose consumption was decreased significantly compared with the normal group (*p* < 0.001), indicating that a stable insulin resistance model was induced successfully.

**Figure 3:**
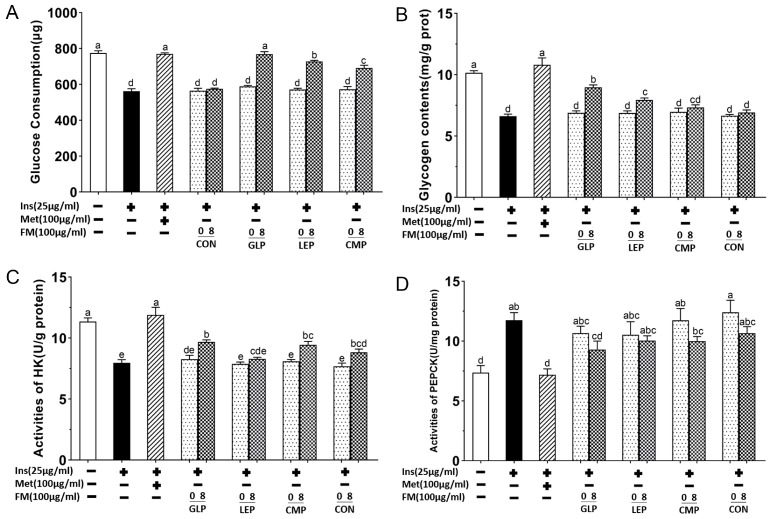
The glucose consumption (**A**), glycogen content (**B**), activities of HK (**C**), and activities of PEPCK (**D**) of HepG2 cells in different groups. + represents that the substance had been added, − represents that the substance had not been added. Values are expressed as mean ± SD (n = 3). Different letters in bars indicate a significant difference (*p* < 0.05).

To evaluate the effects of fermentation supernatants on improving insulin resistance, IR-HepG2 cells were incubated with a cell culture medium for 24 h, which contained 100 μg/mL of different fermentation supernatants (GLP-0, LEP-0, CMP-0, CON-0, GLP-8, LEP-8, CMP-8, CON-8). In addition, IR-HepG2 cells were incubated with a cell culture medium containing 100 μg/mL of metformin for 24 h as a positive control. As shown in Figure 3A, the glucose consumption of fermentation supernatant groups increased to varying degrees compared with the model group. Among them, the glucose consumption in GLP-8, LEP-8, and CMP-8 groups was significantly increased (*p* < 0.05), among which the effect of GLP-8 was almost equivalent to metformin. A comparison of the effects of different fermentation times on glucose consumption showed that, except for the CON-8 group, fermentation for 8 h improved glucose consumption significantly better than fermentation for 0 h.

#### 3.1.3. Glycogen Content, Activities of HK, and PEPCK in IR-HepG2 Cell Model

The glucose metabolism pathways of liver cells include glycolysis, glycogen synthesis and gluconeogenesis [19]. Hexokinase (HK) and phosphoenolpyruvate carboxylase kinase (PEPCK) are the two rate-limiting enzymes in glycolysis and gluconeogenesis, respectively. To explore the effect of fermentation supernatants on these metabolic pathways, glycogen content and activities of HK and PEPCK in IR-HepG2 cells were measured and displayed in Figure 3. As shown in Figure 3B, the glycogen content of the model group was significantly lower than that of the normal group (*p* < 0.05), while this trend was restored to varying degrees after the treatment of polysaccharide fermentation supernatants fermented for 8 h. Specifically, the glycogen content of GLP-8 and LEP-8 groups were significantly higher than that of the model group (*p* < 0.05) and increased by 35.34% and 19.83%, respectively, while the glycogen content of CMP-8 increased by 10.64%, which was not significantly different from the model group. As shown in Figure 3C,D, the HK activity of the model group was significantly lower than that of the normal group. Except for the LEP-8 group, the HK activities were significantly restored after the treatment of fermentation supernatants fermented for 8 h (*p*  <  0.05). However, these groups have no significant difference in the recovery effects of HK activities (*p* > 0.05). Similarly, the PEPCK activity of the model group was significantly higher than that of the normal group, and the intervention of the supernatant from the 8 h polysaccharide fermentation groups restored this trend to varying degrees (*p* > 0.05), while the GLP-8 group was significantly lower than that of the model group (*p*  <  0.05). The results indicated that different polysaccharide fermentation supernatants can have varying degrees of effects on glycogen synthesis and the activities of HK and PEPCK in IR-HepG2 cells. In general, the hypoglycemic effects of fermentation supernatants were GLP-8 > LEP-8 > CMP-8 > CON-8.

#### 3.1.4. Effect of Fermentation Supernatant on mRNA Expression of IRS-1/PI3K/Akt Signaling Pathway

**Figure 4:**
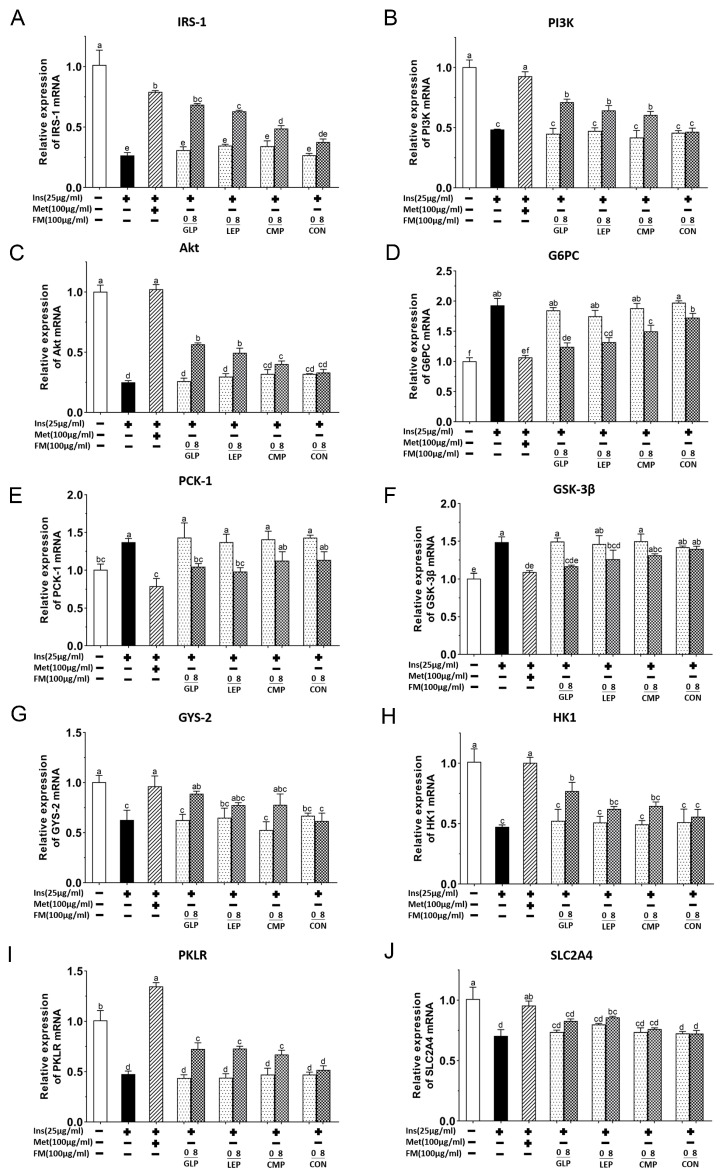
The expression levels of IRS-1/PI3K/Akt pathway-related mRNA in HepG2 cells. + represents that the substance had been added, − represents that the substance had not been added. The mRNA levels of IRS-1 (**A**), PI3k (**B**), Akt (**C**), G6PC (**D**), PCK-1 (**E**), GSK3β (**F**), GYS-2 (**G**), HK1 (**H**), PKLR (**I**), SLC2A4 (**J**). Values are expressed as mean ± SD (n = 3). Different letters in bars indicate a significant difference (*p* < 0.05).

Blockage of insulin receptor-related signaling processes is an important cause of insulin resistance. Studies have shown that the IRS-1/PI3K/Akt signaling pathway is a key pathway for insulin signaling. In order to investigate the hypoglycemic mechanism of fermentation supernatants, RT-qPCR analysis was used to detect the expression levels of IRS-1/PI3K/Akt pathway-related mRNA in IR-HepG2. As shown in Figure 4A–C, compared with the normal group, the mRNA expression levels of IRS-1, PI3K and Akt in the model group were significantly reduced (*p* < 0.05). However, the treatment with Met, GLP-8, LEP-8, and CMP-8 all caused remarkable restoration of these mRNA expressions in contrast with the model group (*p* < 0.05). The recovery effects of fermentation supernatants were GLP-8 > LEP-8 > CMP-8, among which the GLP-8 group had the best recovery effect, with IRS-1, PI3K, and Akt mRNA levels enhanced by 157.50%, 46.97%, and 126.02%, respectively.

G6Pase and PEPCK are the key rate-limiting enzymes in the conversion of noncarbohydrate precursors to glucose during gluconeogenesis [20]. As shown in Figure 4D,E, compared with the normal group, the mRNA expression levels of G6PC and PCK-1 in the model group were increased significantly (*p* < 0.05). However, the treatment with Met, GLP-8 and LEP-8 all caused significant recovery of these mRNA expressions compared with the model group (*p* < 0.05), while CMP-8 only caused significant recovery of G6PC mRNA expression. Overall, the recovery effects of fermentation supernatants were GLP-8 > LEP-8 > CMP-8. Concretely, the mRNA levels of G6PC and PEPCK in the GLP-8 group were reduced by 35.65% and 23.77%, respectively.

GS is the main rate-limiting enzyme for glycogen synthesis [20]. As shown in Figure 4F, compared with the normal group, the mRNA expression level of GSK3β in the model group increased significantly (*p* < 0.05). The decrease of GSK3β mRNA expression in Met, GLP-8, LEP-8 groups were 26.67%, 21.51%, and 15.34%, respectively, which showed obvious differences from the model group (*p* < 0.05). A decrease of GSK3β mRNA expression can activate glycogen synthase (GS), promoting increased glycogen synthesis, and then achieve the effect of lowering blood sugar. As shown in Figure 4G, compared with the model group, the enhancements of GYS2 mRNA expression in the Met, GLP-8, LEP-8 and CMP-8 groups were 53.78%, 42.09%, 23.79%, and 24.32%. Among them, treatment with Met and GLP-8 both caused significant recovery of GYS2 mRNA expression (*p* < 0.05). Treatment with LEP-8 and CMP-8 could also restore GYS2 gene expression but showed no obvious difference from the model group (*p* > 0.05). The restoration effects on GYS2 expression were GLP-8 > LEP-8 > CMP-8.

Hexokinase (HK) and pyruvate kinase (PK) are the key enzymes in glycolysis. As illustrated in Figure 4H,I, significantly lower expression levels of HK1 and PKLR were found in the model group compared with the normal group (*p* < 0.05). After supplementation with Met and GLP-8, the expression level of HK1 was observably increased by 111.56% and 62.36% (*p* < 0.05), respectively. Moreover, the enhancements of PKLR expression level in the Met, GLP-8, LEP-8, and CMP-8 groups were 183.20%, 52.39%, 53.43%, and 40.91%, respectively, which showed obvious differences from the model group (*p* < 0.05).

GLUT4 is essential for glucose transport [21]. As illustrated in Figure 4J, the mRNA expression level of SLC2A4 in the model group was significantly lower than that in the normal group (*p* < 0.05), while the trend was significantly restored by the treatment with Met and LEP-8 (*p* < 0.05). Specifically, the expression level of SLC2A4 was increased by 35.70% and 21.77% in the Met and LEP-8 groups, respectively. In addition, GLP-8, CMP-8, LEP-0, GLP-0, and CMP-0 caused similar trends to the Met and LEP-8 groups in the mRNA expression level, but no significant difference was observed between the model group and the appeal groups. In general, the recovery effects of the polysaccharide fermentation supernatant groups on the expression levels of genes related to the IRS-1/PI3K/Akt pathway were GLP-8 > LEP-8 > CMP-8, which is similar to the trend of glucose consumption and glycogen synthesis.

### 3.2. Effects of Polysaccharides on Fecal Microbiota Composition

#### 3.2.1. Diversity Analysis of Fermentation Supernatant Microbiota

After fermentation, 16S rRNA sequencing was performed to compare the differences in bacterial composition between the fermentation groups. A total of 1,313,708 effective sequences were identified after processing the raw sequence reads using the Illumina MiSeq platform by Shanghai Majorbio Bio-pharm Technology Co., Ltd. It should be noted that the OR group represents the compositions of human feces homogenate prior to fermentation.

Alpha diversity estimators of all groups are listed in Table 2. Shannon, Ace, and Chao index were considerably higher in the GLP-8 group compared with the other groups, while the LEP-8 group showed a higher Shannon index and a lower Simpson index compared with the CON-8 group. In contrast, the CMP-8 group exhibited a significant reduction in the Shannon, Ace, and Chao indexes and an increase in the Simpson index. Alpha diversity analysis showed that different polysaccharides have different effects on the abundance and diversity of intestinal microbiota. Among them, GLP-8 and LEP-8 were more effective in protecting the richness and diversity of the microbiota.

**Figure 5:**
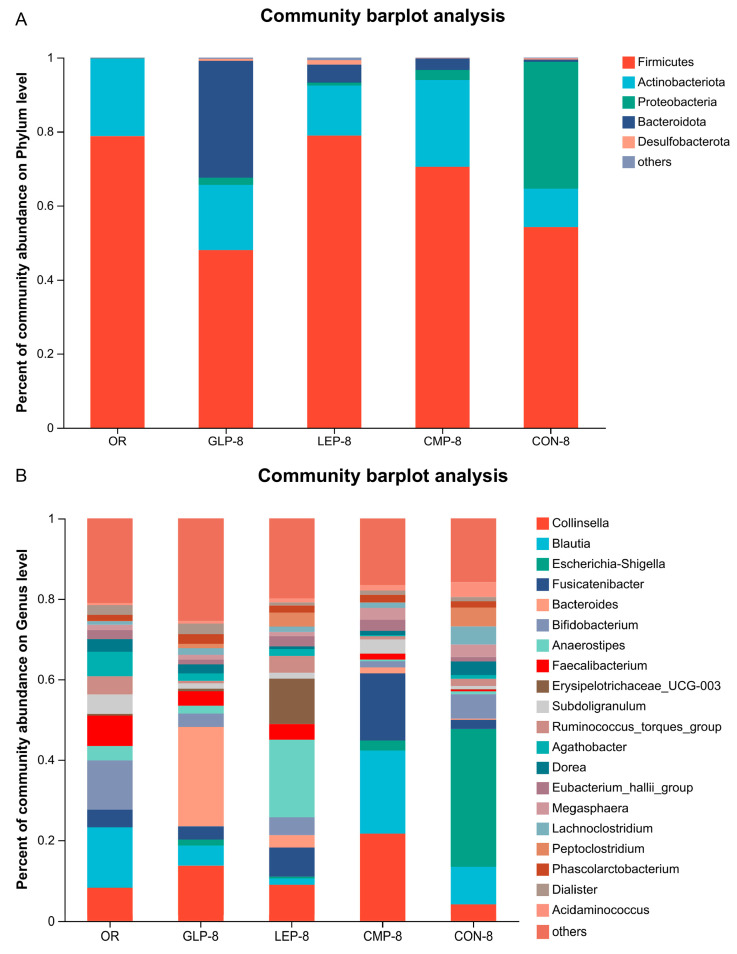

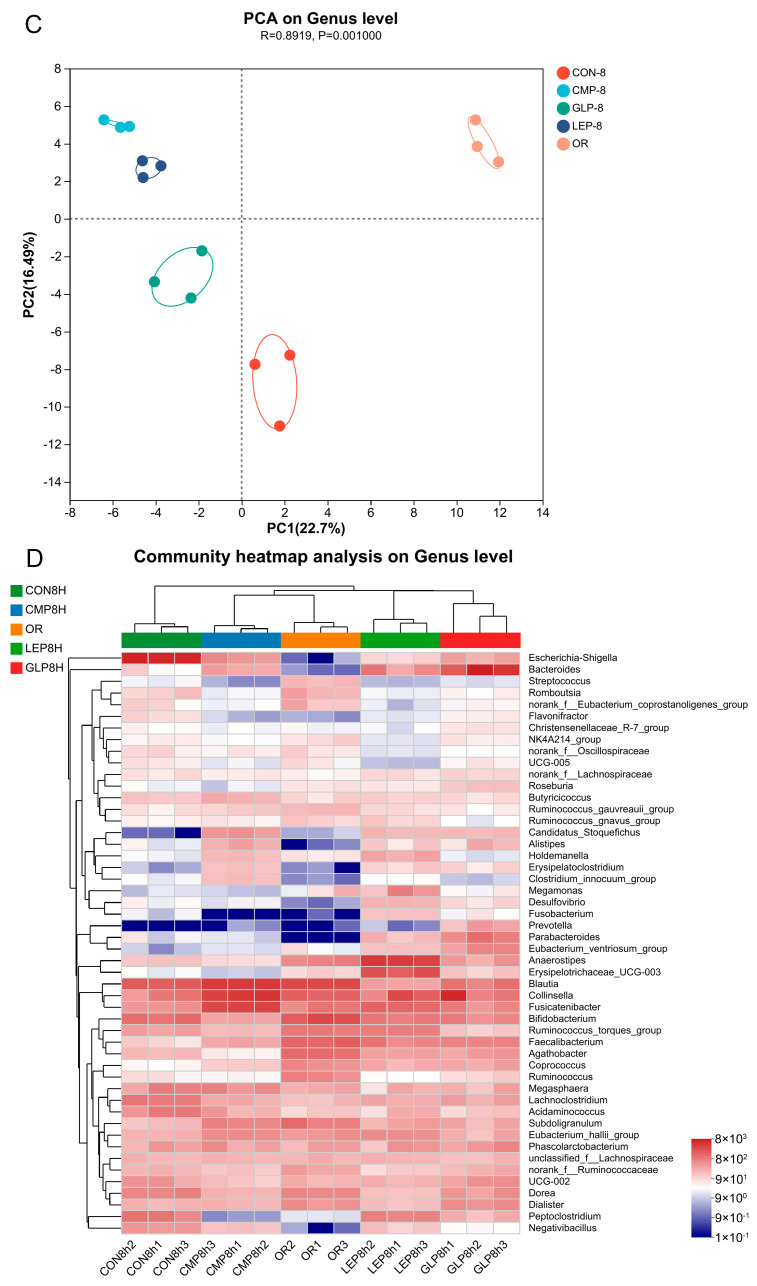
The profile of bacterial composition in fermentation groups, including OR, GLP−8, LEP−8, CMP−8, and CON−8. (**A**) Community composition analysis at the phylum level. (**B**) Community composition analysis at the genus level. (**C**) PCA analysis of genus−level fermentation broth bacteria. (**D**) Community heatmap of fermentation broth flora on genus level.

PCA analysis based on the genus level was analyzed to explore the similarities or differences in community composition between different groups (Figure 5C). The results showed that there were significant differences in the composition of the microbiota between different groups, while the composition of the microbial community after fermentation was significantly different from the OR group.

#### 3.2.2. Composition of Fermentation Supernatant Microbiota

The taxa with an overall abundance greater than 0.1% were summarized. At the phylum level, the bacteria were mainly classified into *Firmicutes*, *Actinobacteria*, *Proteobacteria*, and *Fusobacterium*, among which *Firmicutes* were predominant (Figure 5A). Compared to the CON-8 group, the polysaccharide fermentation groups (GLP-8, LEP-8, and CMP-8) showed variable ascension in the relative abundance of *Actinobacteria* and *Bacteroidota* but a significant decrease in *Proteobacteria*. The top 20 bacteria in relative abundance were analyzed at the genus level (Figure 5B), and the result showed that the relative abundances of *Escherichia-Shigella*, *Dorea*, and *Acidaminococcus* were significantly lower in polysaccharide fermentation groups (GLP-8, LEP-8, and CMP-8) compared with the CON-8 group (*p* < 0.05). On the contrary, the RAs of *Bacteroides*, *Collinsella*, *Fusicatenibacter*, *Faecalibacterium*, *Subdoligranulum*, *Candidatus_Stoquefichus*, and *Eubacterium_hallii_group* in polysaccharide fermentation groups (GLP-8, LEP-8, and CMP-8) showed varying degrees of increase compared with the CON-8 group.

Cluster heatmaps on the genus level were drawn to study the community composition visually. The graph showed that the abundance of *Alistipes* and *Coprococcus* in the polysaccharide fermentation groups (GLP-8, LEP-8, and CMP-8) was significantly higher than those in other groups (Figure 5D). *Roseburia*, *Faecalibacterium* and *Eubacterium_ventriosum_group* were significantly enriched in the GLP-8 and LEP-8 groups (*p* < 0.05). In addition, different bacteria were enriched after the fermentation of different polysaccharides. The abundance of *Prevotella*, *Parabacteroides*, and *Bacteroides* was the highest in the GLP-8 group (*p* < 0.05). The abundance of *Anaerostipes* and *Erysipelotrichaceae_UCG-003* in the LEP-8 group was significantly higher than those in the other groups (*p* < 0.05). The abundance of *Clostridium_innocuum_group* and *Subdoligranulum* in the CMP-8 group was significantly higher than those in the other groups (*p* < 0.05). At the same time, CON-8 group enriched *Escherichia-Shigella*, *Dorea*, *Romboutsia*, and *Acidaminococcus* significantly (*p* < 0.05).

#### 3.2.3. Linear Discriminant Analysis Effect Size

**Figure 6:**
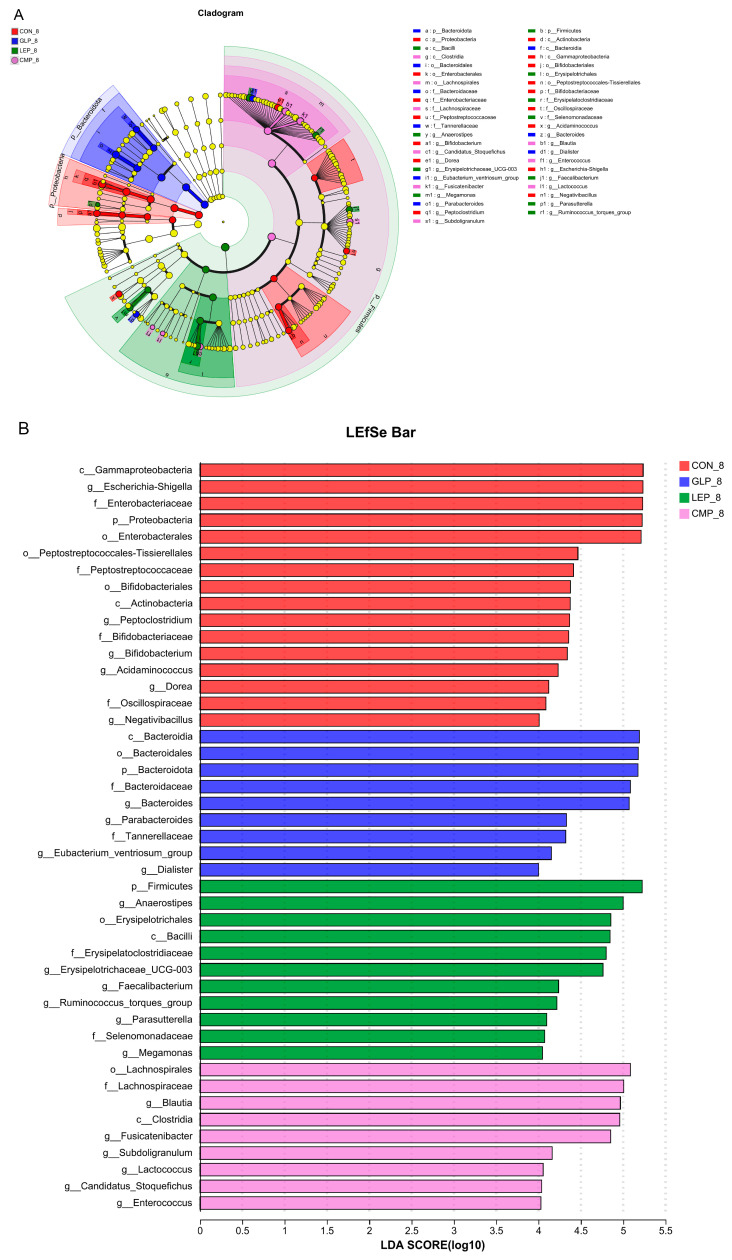
(**A**) The cladogram is according to the LEfSe analysis from the phylum to genus level. (**B**) Histogram of the LDA scores at the genus level from phylum level to genus level, only taxa meeting an LDA significant threshold >4 are shown.

In order to identify changes in specific bacterial microbiota, linear discriminant analysis effect size (LEfSe) was used to compare the microbiota composition of the CON-8, GLP-8, LEP-8 and CMP-8 groups (Figure 6A,B). The analysis was performed with the LDA score > 4.0. At the genus level, *Bacteroides*, *Parabacteroides*, and *Eubacterium_ventriosum_group* were dominant in the GLP-8 group. *Anaerostipes*, *Erysipelotrichaceae_UCG-003*, *Ruminococcus_torques_group*, *Faecalibacterium*, and *Megamonas* were dominant in the LEP-8 group. *Blautia*, *Fusicatenibacter*, *Subdoligranulum*, and *Candidatus_Stoquefichus* were dominant in the CMP-8 group. In addition, *Escherichia-Shigella*, *Peptoclostridium*, *Bifidobacterium*, *Acidaminococcus*, and *Dorea* dominated the CON-8 group. These results indicate that different polysaccharides can up-regulate the abundance of some bacteria, such as *Alistipes*, after fermentation and enrich different bacteria, respectively.

### 3.3. Metabolite Profiles between Different Fermentation Groups

#### 3.3.1. Effect of Polysaccharides on Metabolites

**Figure 7:**
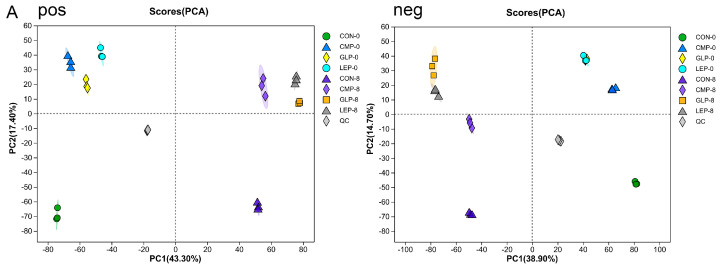

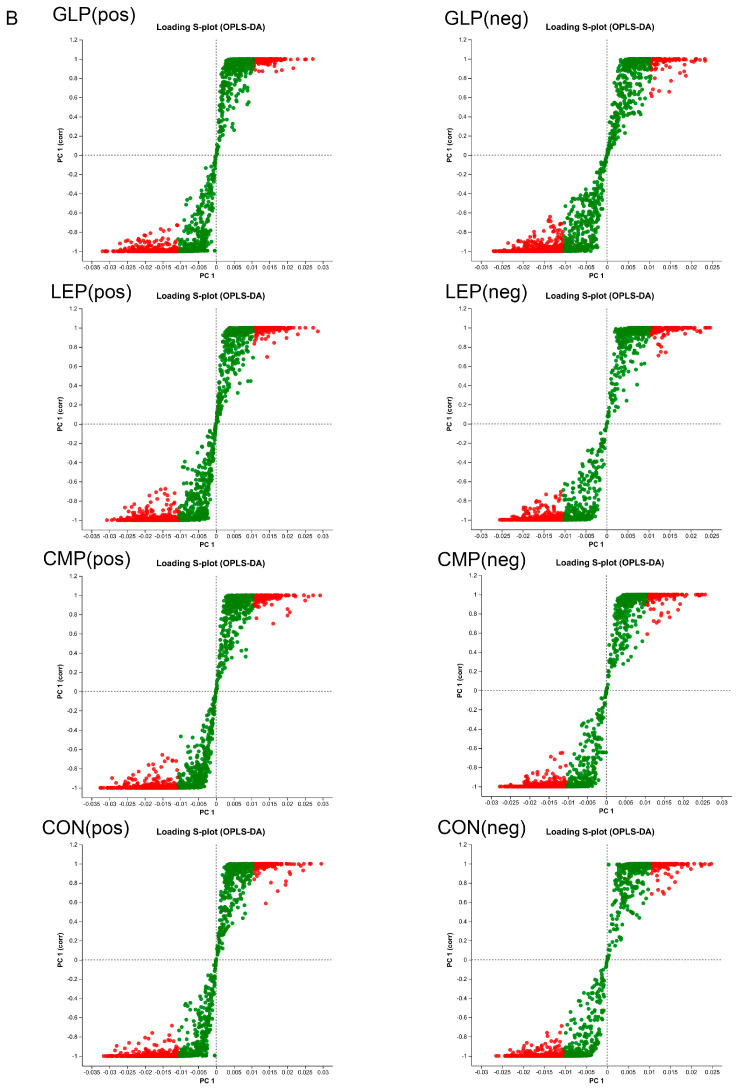

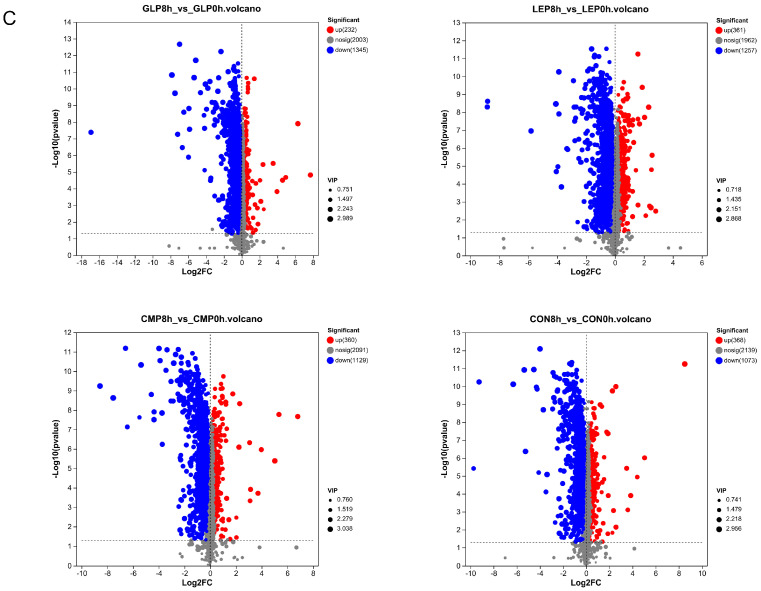

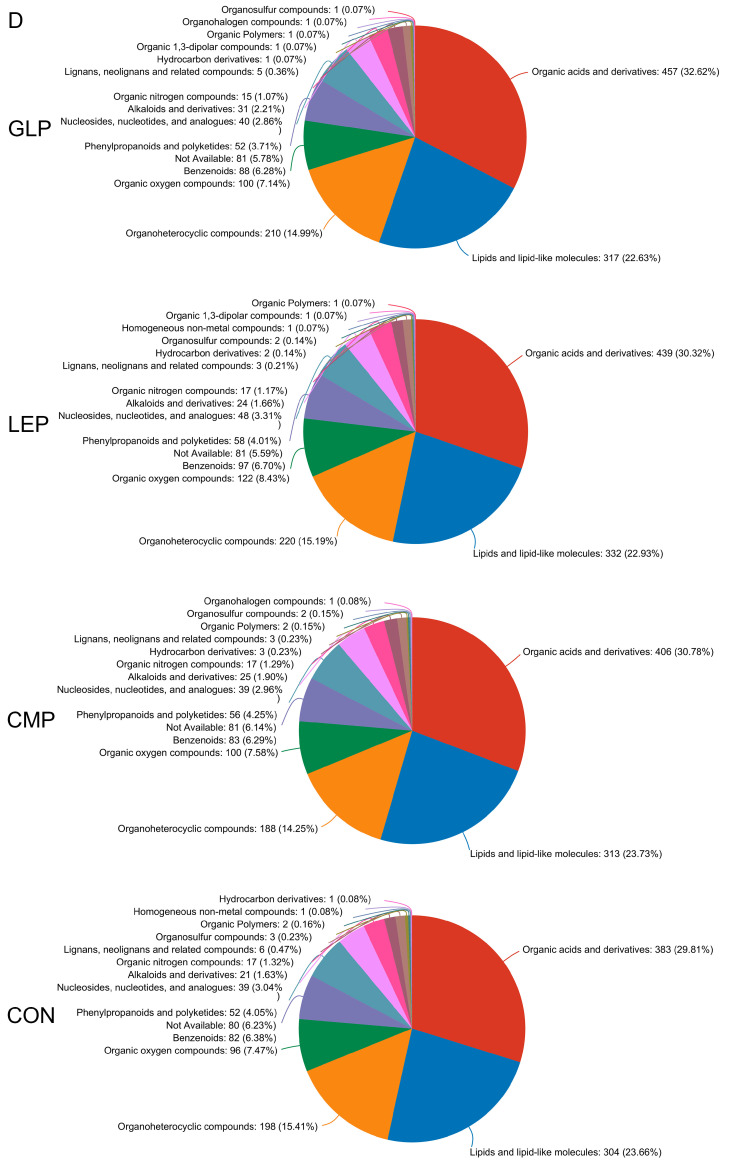
Profiles of microbial metabolites in fermentation and non-fermentation groups, including GLP−8, LEP−8, CMP−8, CON−8, GLP−0, LEP−0, CMP−0, and CON−0. (**A**) PCA analysis of polysaccharides fermentation and non-fermentation metabolites in both positive (pos) and negative (neg) ion modes. (**B**) S-plots in OPLS-DA of polysaccharides fermentation metabolites in both positive (pos) and negative (neg) ion modes. The red dot represents the metabolite with a VIP value ≥ 1 while the green dot represents the metabolite with a VIP value < 1. (**C**) Volcano plots to analyze the profile of the differential metabolites between polysaccharides fermentation and non-fermentation metabolites. (**D**) Pie charts of the differential metabolites between polysaccharides fermentation and non-fermentation metabolites based on the HMDB chemical taxonomy (superclass).

According to the above results, there were differences in the hypoglycemic activities with different carbon sources and different fermentation times. As shown in Figure 7A, principal component analysis (PCA) performed in positive and negative ion modes showed considerable separation between the 0 h and 8 h fermentation groups, indicating that metabolites changed significantly during fermentation. In the meantime, the polysaccharide fermentation groups (GLP-8, LEP-8, and CMP-8) are relatively close to each other and far away from the CON-8 group, illustrating that the metabolite compositions of these three groups have certain similarities.

#### 3.3.2. Screening of Differential Metabolites

The S-plots generated through OPLS-DA illustrated the contribution of the distributed metabolites. As shown in the O-PLS-DA S-plot generated (Figure 7B), significant differences in the distribution of points at the end of the S-plot generated were obtained for 8 h fermentation groups and 0 h fermentation groups either the positive or negative ion mode, showing that the metabolites of each group had changed significantly after 8 h of fermentation. To examine the quantitative information of differential metabolites between groups, the differential metabolites with *p* < 0.05, VIP > 1 were screened from all metabolites using the volcano plot (Figure 7C). After 8 h of fermentation, 1441, 1577, 1618, and 1489 differential metabolites were found in the CON, GLP, LEP, and CMP groups, respectively. These metabolites were further classified according to the Human Metabolome Database (HMDB) compound classification analysis (Figure 7D) and were mainly annotated as Organic acids and derivatives, Lipids and lipid-like molecules, Organoheterocyclic compounds, Organic oxygen compounds, etc. Preliminary experiments found that the polysaccharide fermentation supernatants had hypoglycemic effects, and the effects of the GLP and LEP groups were better. In order to screen out metabolites with potential hypoglycemic effects, we focused on differential metabolites that were significantly up-regulated in the polysaccharide groups but not significantly up-regulated in the CON group, especially those with higher abundance in the GLP and LEP groups. Among them, 22 metabolites were found to have potential hypoglycemic effects (Table 3).

#### 3.3.3. Specific Gut Bacteria and Metabolites May Contribute to the Hypoglycemic Effects of IR-HepG2 Cells

**Figure 8:**
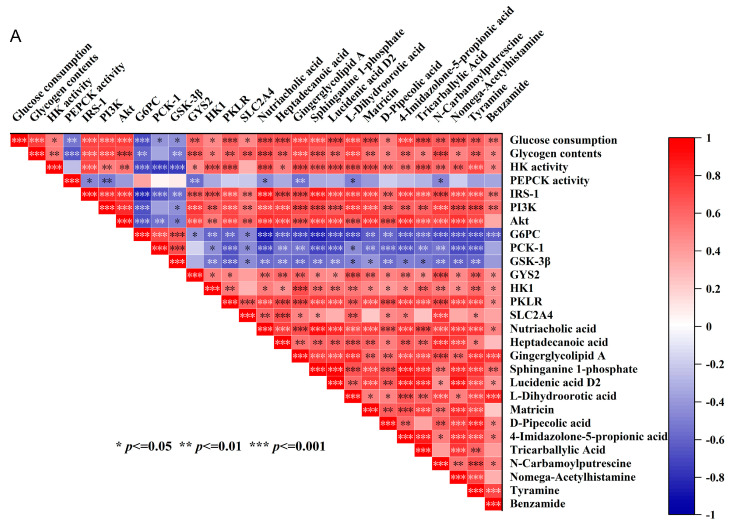

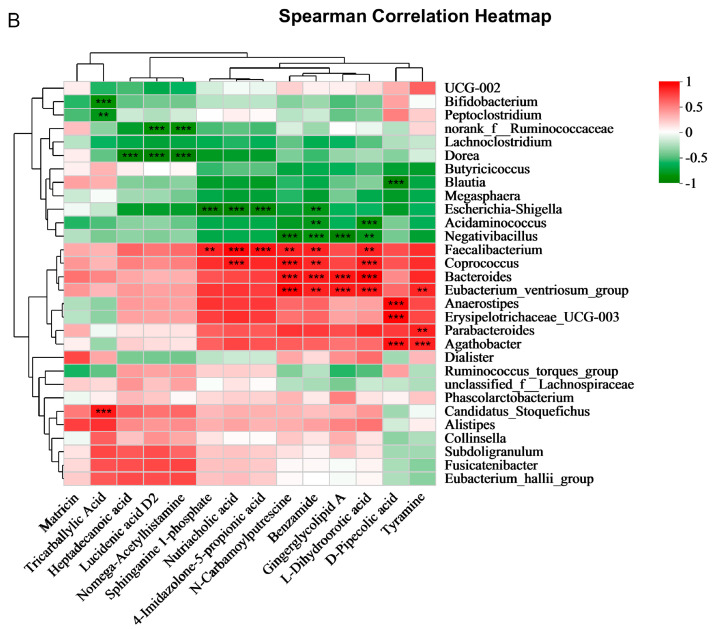
(**A**) Spearman correlation analysis of hypoglycemic activity−related indicators and potential key metabolites reflecting the hypoglycemic activity (|R| > 0.50, *p* < 0.05). (**B**) Spearman correlation Heatmap of top 30 abundant bacteria and potential key metabolites (|R| > 0.80, *p* < 0.05). ‘*’ indicates *p* < 0.05, ‘**’ indicates *p* < 0.01, and ‘***’ indicates *p* < 0.001.

To further screen potential key metabolites related to the hypoglycemic activities of polysaccharides, spearman correlation analysis was performed on the hypoglycemic activity-related indicators measured in the previous experiment and the abundance of 22 differential metabolites obtained through the preliminary screening. As shown in Figure 8B, 14 metabolites were significantly related to the hypoglycemic activity-related indicators according to the Spearman correlation analysis (|R| > 0.50, *p* < 0.05). Specifically, Nutriacholic acid, gingerglycolipid A, L-Dihydroorotic acid, and N-carbamoylputrescine had the strongest correlation with various hypoglycemic activity-related indicators related to hypoglycemic activity and mRNA expression related to the IRS-1/PI3K/Akt pathway. Heptadecanoic acid, sphinganine 1-phosphate, Lucidenic acid D2, matricin, 4-Imidazolone-5-propionic acid, N-carbamoylputrescine and tyramine were substantially and positively correlated to glucose consumption, glycogen content, HK activity, IRS-1, PI3K, and AKT levels. D-Pipecolic acid had a strong positive correlation with PKLR and a strong negative correlation with PCK-1. Benzamide showed a strong negative correlation with G6PC.

To further analyze the key bacteria potentially responsible for producing metabolites related to hypoglycemic effects, the relationship between the above 14 metabolites and the top 30 abundant bacteria was also analyzed using Spearman’s correlation analysis (Figure 8B). A total of 17 bacterial species were found to be significantly correlated with these metabolites’ abundance (|R| > 0.80, *p* < 0.05). Among them, the abundance of tricarballylic acid was substantially and positively correlated to the abundance of *Candidatus_Stoquefichu* and was negatively correlated to the abundance of *Bifidobacterium* and *Peptoclostridium*. Heptadecanoic acid, Lucidenic acid D2, and nomega-acetylhistamine presented a strong negative correlation with *norank_f_Ruminococcaceae* and *Dorea*. Sphinganine 1-phosphate, Nutriacholic acid, and 4-Imidazolone-5-propionic acid showed strong positive correlations with *Faecalibacterium* and *Coprococcus* but strong negative correlations with *Escherichia-Shigella*. N-carbamoylputrescine, benzamide, gingerglycolipid A, and L-Dihydroorotic acid were positively correlated to the *Faecalibacterium*, *Coprococcus*, *Bacteroides*, *Eubacterium_ventriosum_group*, but negatively correlated to *Acidaminococcus* and *Negativibacillus*. D-Pipecolic acid showed a strong positive correlation with *Erysipelotrichaceae_UCG-003*, *Agathobacter*, and *Anaerostipes*, while it showed a strong negative correlation with *Blautia*. The level of tyramine is closely related to the abundance of *Eubacterium_ventriosum_group*, *Parabacteroides*, and *Agathobacter*. These bacteria may be key bacteria in the production of potential key metabolites with hypoglycemic effects.

### 3.4. Poria Cocos Alkaline-Soluble Polysaccharides’ Interventions Verified the Hypoglycemic Effect of Key Bacteria and Metabolites

To verify the potential key metabolites and bacteria for alleviating diabetes, the *Poria cocos* alkaline-soluble polysaccharide was used for in vitro fermentation, and the fermentation supernatants were used to intervene in IR-HepG2 cells.

**Figure 9:**
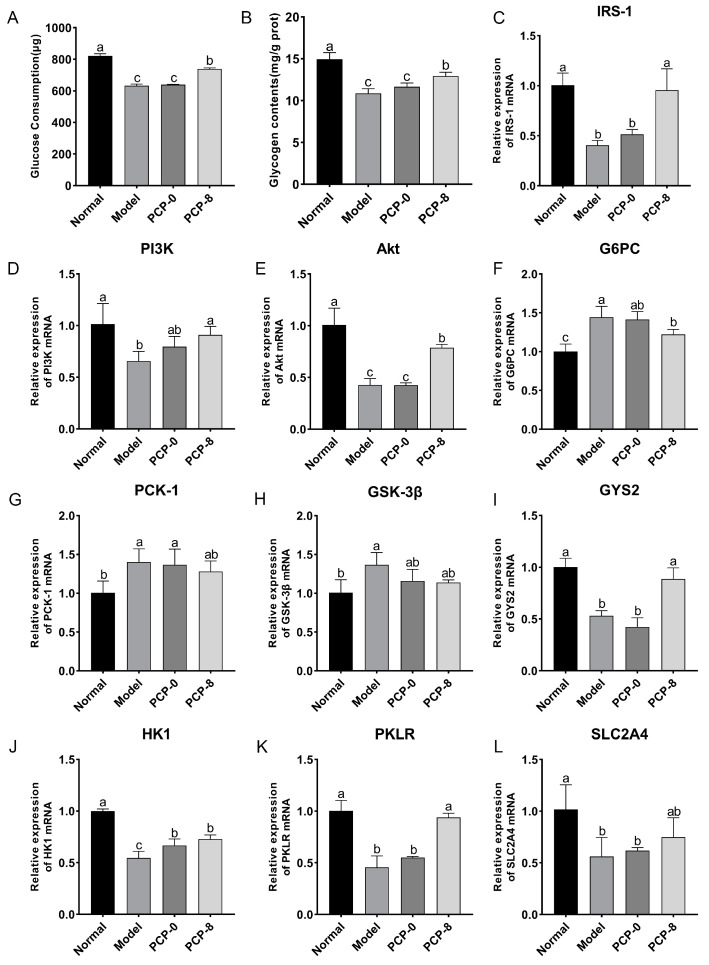
Levels of hypoglycemic activity-related indicators in the PCP-0 and PCP-8 groups. The glucose consumption (**A**), glycogen content (**B**), and expression levels of IRS-1/PI3K/Akt pathway-related mRNA in HepG2 cells (**C**–**L**). Values are expressed as mean ± SD (n = 3). Different letters in bars indicate a significant difference (*p* < 0.05).

As shown in Figure 9A,B, the glucose consumption and glycogen synthesis of the PCP-8 group were significantly higher than those of the model group (*p* < 0.05), while the glucose consumption and glycogen synthesis of the PCP-0 group were not significantly different from those of the model group. After intervention with the fermentation supernatants, the expression of genes related to the IRS-1/PI3K/Akt pathway was further evaluated (Figure 9C–L). Our results showed that the expression levels of the genes in the PCP-8 group were significantly restored compared with the model group except for SLC2A4, PCK-1, and GSK-3β. The HK-1 gene expression level in the PCP-0 group was significantly restored, while the expression levels of other genes were similar to the model group. Therefore, we propose that the hypoglycemic activity of *Poria cocos* alkali-soluble polysaccharide had been improved after fecal fermentation.

To analyze the reasons for the difference in hypoglycemic activity before and after fermentation, non-targeted metabolomic analysis was performed on the fermentation supernatants of the PCP-8 group and PCP-0 group. As shown in Table 4, the levels of eight metabolites were significantly increased in the PCP-8 group compared with the PCP-0 group among the 14 potential key metabolites, including gingerglycolipid A, sphinganine 1-phosphate, matricin, tricarballylic Acid, N-carbamoylputrescine, nomega-acetylhistamine, tyramine, and benzamide. In addition, compared with the PCP-0 group, the level of Nutriacholic acid, Lucidenic acid D2, L-Dihydroorotic acid, D-Pipecolic acid, and 4-Imidazolone-5-propionic acid in the PCP-8 group increased with no significant difference, and the level of Heptadecanoic acid decreased, but there was no significant difference. To clarify which intestinal bacteria are enriched during the fermentation process of *poria cocos* alkali-soluble polysaccharide, the bacterial community structure of the PCP-8 group was compared with the bacterial community structure of the CON-8 group. As shown in Table 5, bacteria related to the synthesis of eight differential metabolites with significantly increased levels in the PCP-8 group, including *Faecalibacterium*, *Coprococcus*, *Bacteroides*, *Eubacterium_ventriosum_group*, *Anaerostipes*, and *Candidatus_Stoquefichus* were significantly enriched in the PCP-8 group. The abundance of bacteria that were negatively correlated with the levels of these metabolites, including *Escherichia-Shigella*, *Acidaminococcus*, and *Negativibacillus* was significantly reduced in the PCP-8 group.

## 4. Discussion

In this research, we investigated the hypoglycemic activity of metabolites produced by in vitro fermentation of four different edible fungi polysaccharides (including *Ganoderma lucidum*, *Cordyceps militaris*, *Lentinula edodes*, and *Poria cocos*) by human fecal bacteria. Using IR-HepG2 cells as a model, we found that the metabolites from fermented polysaccharides can significantly increase the glucose consumption and glycogen synthesis of IR-HepG2. Additionally, the metabolites up-regulated the level of HK and down-regulated the level of PEPCK. All four fermentation polysaccharides restored the expression levels of genes associated with the IRS-1/PI3K/Akt signaling pathway to varying degrees. Among the metabolites of fermented polysaccharides, *Ganoderma lucidum* and *Lentinula edodes* had better hypoglycemic effects. We further analyzed and validated the specific metabolites potentially associated with hypoglycemic effects and bacteria that may produce these metabolites. A series of metabolites, such as gingrglycolipid A, sphinganine 1-phosphate, matricin, etc., were proposed as potential biomarkers to evaluate the hypoglycemic effect of different polysaccharides.

Many edible fungi polysaccharides, including *Ganoderma lucidum*, *Lentinus edodes*, and *Cordyceps militaris*, have been reported to have hypoglycemic effects [22,23,24], while these researches mainly focus on the activities of polysaccharides themselves. Some studies have compared the hypoglycemic activities of polysaccharides from different sources, but the reasons for the differences have not been clarified. It was only speculated that the reasons may be related to the differences in physical and chemical properties of polysaccharides [25,26,27]. The structure of polysaccharides will be modified and transformed after fermentation by intestinal bacteria, which will affect their biological functions. In addition, different polysaccharides show significant differences in monosaccharide composition, molecular weight, glycosidic bonds, conformation, etc., making it difficult to compare the hypoglycemic activity based on their physical and chemical properties [28]. However, all polysaccharides can’t be directly absorbed to make a role in the human or mammalian small intestine but exert their activity after microbiota fermentation in the large intestine. Therefore, it is feasible to quickly evaluate the hypoglycemic activity of different polysaccharides based on the enriched fermentation metabolites. To our knowledge, this is the first report to study and compare the hypoglycemic effects of metabolites from the in vitro fermentation of various edible fungi polysaccharides using human fecal bacteria.

We found that the levels of some metabolites increased significantly with increasing hypoglycemic activity. Most of these metabolites have beneficial effects on alleviating diabetes through various pathways. For example, gingrglycolipid A was found to be the main hypoglycemic active ingredient of mulberry leaf extract [29]. Sphinganine 1-phosphate inhibits glucose production in the liver and improves insulin sensitivity by suppressing inflammatory actions of macrophages [30]. Matricin had been reported to have potential anti-inflammatory effects and was found to be one of the bioactive components of *Phellinus linteus* crude polysaccharide in improving diabetes [31,32]. Tricarballylic acid may improve diabetes by regulating inflammatory responses [33]. N-carbamoylputrescine, like SCFAs, is an important intestinal growth factor that helps maintain intestinal homeostasis [34]. Nomega-acetylhistamine was found to be significantly increased in T2DM rats after treatment with Danggui Buxue decoction [35]. Tyramine and its derivatives have powerful α-glucosidase inhibitory effects and may serve as competing amine oxidase substrates to improve diabetes [36]. Benzamide was found to improve diabetes by inhibiting glycogen phosphorylase (GP) and activating glucokinase (GK) [37]. All eight metabolites were significantly associated with the hypoglycemic effect of polysaccharide fermentation supernatants. Therefore, these eight metabolites may be potential key metabolites to assess the hypoglycemic activity of edible fungi polysaccharides from different sources.

These differential metabolites were generated from human feces’ in vitro fermentation of polysaccharides. Most of the dominant bacterial genera in fermented GLP, LEP and CMP, such as *Alistipes*, *Coprococcus*, *Faecalibacterium*, and *Eubacterium_ventriosum_group*, etc., were reported to be closely related to the hypoglycemic effects [38,39,40,41]. *Bacteroides*, which were significantly enriched in fermented GLP, play an important role in regulating the synthesis of short-chain fatty acids [42]. *Parabacteroides* can indirectly alleviate diabetes by producing indoleacrylic acid [43]. *Anaerostipes* and *Megamonas*, which were significantly enriched in the fermented LEP, were reported to be significantly related to the synthesis of acetic acid and propionic acid [44,45]. *Candidatus_Stoquefichus*, significantly enriched in the fermented CMP, was reported to have potential anti-inflammatory effects [46]. It is worth noting that our results showed that intestinal microbiota was responsible for eight potential key metabolites closely related to hypoglycemic activity, including gingrglycolipid A, sphinganine 1-phosphate, etc. However, few studies have reported the specific genus of bacteria involved in these metabolites. Our results of correlation analysis revealed that they were associated with the abundance of *Candidatus_Stoquefichu*, *Faecalibacterium*, *Coprococcus*, *Bacteroides*, *Eubacterium_ventriosum_group*, *Erysipelotrichaceae_UCG-003*, *Anaerostipes*, *Parabacteroides*, and *Agathobacter*. Most of these bacteria were significantly enriched in the fermented PCP. This result proves that these eight metabolites should be potential key metabolites reflecting the hypoglycemic efficacy of edible fungi polysaccharides.

The mRNA expressions of the IRS-1/PI3K/Akt signaling pathway were explored to further reveal the potential mechanism of the hypoglycemic effects of metabolites. Correlation analysis showed that the levels of these metabolites were significantly correlated with the expression of pathway-related mRNAs. One of the important causes of insulin resistance is the loss of the insulin signaling pathway in the liver, while the IRS-1/PI3K/Akt signaling pathway plays an important role in insulin signaling [47]. IRS-1, PI3K, Akt in the liver cells act as a nutrient-responsive brake for insulin signal transduction [48]. Further affects the expression of GSK3β and FOXO1 [49], thus affecting the enzyme reactions of glycogen synthesis and gluconeogenesis. Expression of Akt also affects the glucose transporter named GLUT4, thus accelerating insulin-stimulated glucose transport and inhibiting gluconeogenesis [50]. Among the eight potential key metabolites, sphinganine 1-phosphate is able to protect against the development of hepatic insulin resistance by activating the Akt pathway via the receptor subtypes S1PR1 and/or S1PR3 and by enhancing mitochondrial functions [51]. Benzamide derivative MID-00935 disrupted the molecular interaction of MG53 and IRS-1, abrogated MG53-induced IRS-1 ubiquitination and degradation and improved insulin signaling in C2C12 myotubes [52]. Even though no studies have explored the relationship between the remaining metabolites and IRS-1/PI3K/Akt signaling pathway, the correlation analysis revealed that they were associated with the expression of pathway-related mRNAs. Our results suggest that these metabolites may also exert hypoglycemic effects through the IRS-1/PI3K/Akt pathway. However, further studies should be performed to confirm this possibility.

## 5. Conclusions

In conclusion, our results showed that the fermentation metabolites of *Ganoderma lucidum*, *Lentinus edodes*, *Cordyceps militaris*, and *Poria cocos* alkaline-soluble polysaccharides showed stronger hypoglycemic activity after in vitro fermentation by fecal bacteria. All edible fungi polysaccharides present intestinal probiotic activity and can regulate the bacterial community structure. These polysaccharides enriched some specific metabolites and, therefore, improved their hypoglycemic activity. These specific metabolites, including gingerglycolipid A, sphinganine 1-phosphate, matricin, tricarballylic acid, N-carbamoylputrescine, nomega-acetylhistamine, tyramine, and benzamide, were considered potential key metabolites to evaluate the hypoglycemic effects. Furthermore, their levels were strongly positively correlated with the abundance of *Candidatus_Stoquefichu*, *Faecalibacterium*, *Coprococcus*, *Bacteroides*, *Eubacterium_ventriosum_group*, *Anaerostipes*, *Parabacteroides*, and *Agathobacter*. These metabolites may alleviate insulin resistance in IR-HepG2 cells by regulating the expression levels of genes related to the IRS-1/PI3K/Akt signaling pathway. These findings will help further develop rapid screening methods to evaluate whether unknown polysaccharides have hypoglycemic abilities.

## Figures and Tables

**Table 1:** Sequence of primers used in quantitative real-time PCR.

Gene	Sequence 5′-3′
IRS1-F	GAACTCACTCGGCAGGCACATC
IRS1-R	TGGTGGGTAGGCAGGCATCATC
PI3K-F	GAAGCAGCAACCGAAACAAAGC
PI3K-R	ACCACTACAGAGCAGGCATAGC
Akt-F	AGGATGTGGACCAACGTGAGG
Akt-R	GCAGGCAGCGGATGATGAAG
G6PC-F	GTCTGTCTGTCACGAATCTACCTTG
G6PC-R	ATGCTGTGGATGTGGCTGAAAG
PCK1-F	CCAATGCCATCAAGACCATCCAG
PCK1-R	TCATCAATGCCTTCCCAGTAAACG
GSK3β-F	ACTTCACCACTCAAGAACTGTCAAG
GSK3β-R	TGTCCACGGTCTCCAGTATTAGC
GYS2-F	AACTAACATCACCACCAACGACAG
GYS2-R	CATCCTCCACTTCATCTTCCACATC
HK1-F	GCGGCTTGTGCTGCTCAG
HK1-R	TCTCCACCTGCGACACGAAG
PKLR-F	GAAGGACACGGCATCAAGATCATC
PKLR-R	CTCACCTCCAGGATTTCATCAAACC
SLC2A4-F	TCCAACAGATAGGCTCCGAAGATG
SLC2A4-R	CAAGCACCGCAGAGAACACAG

**Table 2:** Alpha diversity of fermentation broth flora at OTU level.

Group\Estimator	Shannon	Simpson	Ace	Chao
OR	3.77 ± 0.05 ^ab^	0.04 ± 0.00 ^b^	326.96 ± 23.27 ^b^	319.18 ± 28.50 ^b^
GLP-8	3.82 ± 0.28 ^a^	0.07 ± 0.03 ^b^	361.64 ± 17.00 ^a^	366.25 ± 22.29 ^a^
LEP-8	3.55 ± 0.07 ^b^	0.07 ± 0.01 ^b^	335.18 ± 12.14 ^ab^	330.81 ± 9.72 ^b^
CMP-8	3.14 ± 0.12 ^c^	0.11 ± 0.01 ^a^	323.69 ± 6.66 ^b^	316.00 ± 6.47 ^b^
CON-8	3.22 ± 0.02 ^c^	0.13 ± 0.01 ^a^	342.45 ± 6.46 ^ab^	338.92 ± 8.85 ^ab^

Note: Values are expressed as mean ± SD (n = 3). Different letters in bars indicate a significant difference (*p* < 0.05).

**Table 3:** Differential metabolites that may have potential hypoglycemic effects.

Metabolite	Metab ID	Mode	CAS ID	Formula
Nutriacholic acid	metab_10614	neg	4651-67-6	C_24_H_38_O_4_
N-Methyl-1-deoxynojirimycin	metab_2758	pos	69567-10-8	C_7_H_15_NO_4_
Heptadecanoic acid	metab_1745	pos	3546-17-6; 106182-29-0; 506-12-7	C_17_H_34_O_2_
Gingerglycolipid A	metab_6324	pos	145937-22-0	C_33_H_56_O_14_
Sphinganine 1-phosphate	metab_6251	pos	19794-97-9	C_18_H_40_NO_5_P
Lucidenic acid D2	metab_5169	pos	98665-16-8	C_29_H_38_O_8_
L-Dihydroorotic acid	metab_8809	pos	5988-19-2	C_5_H_6_N_2_O_4_
Cyclohexanecarboxylic acid	metab_16424	neg	98-89-5	C_7_H_12_O_2_
Matricin	metab_6723	pos	29041-35-8	C_17_H_22_O_5_
D-Pipecolic acid	metab_1245	pos	1723-00-8	C_6_H_11_NO_2_
Sparfloxacin	metab_5420	pos	110871-86-8	C_19_H_22_F_2_N_4_O_3_
4-Imidazolone-5-propionic acid	metab_1435	pos	17340-16-8	C_6_H_8_N_2_O_3_
Tricarballylic Acid	metab_14608	neg	99-14-9	C_6_H_8_O_6_
Gamma-Eudesmol rhamnoside	metab_14104	neg	349112-31-8	C_21_H_36_O_5_
Ergocornine	metab_7383	pos	564-36-3	C_31_H_39_N_5_O_5_
N-Carbamoylputrescine	metab_1423	pos	6851-51-0	C_5_H_13_N_3_O
Nomega-Acetylhistamine	metab_8547	pos	673-49-4	C_7_H_11_N_3_O
Lycorine	metab_5677	pos	476-28-8	C_16_H_17_NO_4_
N6,N6,N6-Trimethyl-L-lysine	metab_5628	pos	19253-88-4	C_9_H_20_N_2_O_2_
Huperzine b	metab_15873	neg	103548-82-9	C_16_H_20_N_2_O
Tyramine	metab_16366	neg	51-67-2	C_8_H_11_NO
Benzamide	metab_17548	neg	55-21-0	C_7_H_7_NO

**Table 4:** Fourteen potential key metabolites content in the PCP-8 group and the PCP-0 group.

Metabolite	Metab ID	Significant	Regulate	*m*/*z*	PCP-8 Mean	PCP-8 SD	PCP-0 Mean	PCP-0 SD
Nutriacholic acid	metab_10614	no	up	389.2708	7.554	0.01353	7.077	0.01193
Heptadecanoic acid	metab_1745	no	down	288.2891	5.621	0.02393	5.69	0.009962
Gingerglycolipid A	metab_6324	yes	up	641.3563	4.808	0.22	3.899	0.3994
Sphinganine 1-phosphate	metab_6251	yes	up	420.228	6.113	0.04017	5.333	0.04451
Lucidenic acid D2	metab_5169	no	up	537.2494	4.789	0.05297	4.451	0.005533
L-Dihydroorotic acid	metab_8809	no	up	200.0681	4.333	0.07629	4.14	0.05721
Matricin	metab_6723	yes	up	630.3285	5.583	0.1096	4.54	0.232
D-Pipecolic acid	metab_1245	no	up	152.0679	5.621	0.03066	5.447	0.02621
4-Imidazolone-5-propionic acid	metab_1435	no	up	157.0606	4.297	0.1564	3.973	0.005532
Tricarballylic Acid	metab_14608	yes	up	175.0246	4.863	0.02584	3.753	0.1764
N-Carbamoylputrescine	metab_1423	yes	up	173.1395	4.792	0.05204	3.882	0.1446
Nomega-Acetylhistamine	metab_8547	yes	up	154.0973	4.092	0.07222	3.285	0.03266
Tyramine	metab_16366	yes	up	136.0764	4.381	0.1467	3.39	0.08128
Benzamide	metab_17548	yes	up	166.0507	3.711	0.08852	2.713	0.1963

**Table 5:** RAs of bacteria related to the synthesis of eight differential metabolites with significantly increased levels in the PCP-8 group.

Species Name	Significant	Regulate	PCP-8 Mean	PCP-8 Sd	CON-8 Mean	CON-8 Sd
*Bifidobacterium*	no	up	7.044	2.573	6.118	0.8949
*Peptoclostridium*	no	up	7.231	0.5436	4.616	1.311
*norank_f__Ruminococcaceae*	yes	up	1.721	0.06132	1.035	0.1088
*Dorea*	yes	up	4.877	0.5175	3.409	0.4135
*Blautia*	no	down	9.106	1.041	9.34	0.6807
*Escherichia-Shigella*	yes	down	12.64	3.18	34.18	2.083
*Acidaminococcus*	yes	down	0.4363	0.07902	3.766	1.309
*Negativibacillus*	yes	down	1.514	0.1336	2.164	0.1598
*Faecalibacterium*	yes	up	1.186	0.2241	0.4235	0.1428
*Coprococcus*	yes	up	0.3538	0.05773	0.1825	0.02315
*Bacteroides*	yes	up	1.963	0.1521	0.2867	0.2351
*Eubacterium_ventriosum_group*	yes	up	1.088	0.2302	0.03385	0.02429
*Anaerostipes*	yes	up	1.445	0.07625	0.6959	0.05073
*Erysipelotrichaceae_UCG-003*	no	up	0.1617	0.0277	0.1197	0.03493
*Parabacteroides*	no	up	1.465	0.949	0.1544	0.1301
*Agathobacter*	no	up	2.295	0.5811	0.9188	0.08288
*Candidatus_Stoquefichus*	yes	up	0.09542	0.00634	0.003066	0.002658

## Data Availability

The data presented in this study are available on request from the corresponding author. The data are not publicly available due to privacy restrictions.
